# Family systems care approaches and methodologies for maternal, newborn and child health in low- and middle-income countries: a scoping review

**DOI:** 10.1080/16549716.2025.2567714

**Published:** 2025-10-15

**Authors:** Christina Schuler, George Edward Ntow, Rebecca Berner, Salai Thet Naing Oo, Riccardo E. Pfister

**Affiliations:** a Institute of Global Health, Faculty of Medicine, University Geneva, Geneva, Switzerland; b Institute of Nursing, Department of Health Sciences, Zurich University of Applied Sciences, Winterthur, Switzerland; c Health Sciences, Dodowa Health Research Centre, Dodowa, Ghana; d Faculty of Medicine, University of Basel, Basel, Switzerland; e Health Sciences, SPITEX Thurgau Nordwest – Swiss Home Care Services, Diessenhofen, Switzerland; f Neonatal and Pediatric Intensive Care Unit, University Hospitals of Geneva and Geneva University, Geneva, Switzerland

**Keywords:** continuum of care, family-centered care, health personnel, implementation science, implementation strategy, implementation frameworks, health services, cultural adaptation, health outcomes

## Abstract

Integrating family-centered maternal, newborn, and child health (MNCH) services along the care continuum can improve health outcomes in low- and middle-income countries (LMICs). However, family systems care remains underutilized from hospital to community and home. This scoping review examines approaches, methods, and tools for incorporating family systems care into MNCH care in LMICs. Following the recommendations of the Joanna Briggs Institute and the PRISMA ScR guidelines, we adopted a three-step search strategy. It included searches 1) in three databases (Medline, CINAHL, and Web of Science), 2) of the literature on governmental and non-governmental homepages, and 3) in the references of included studies. Published English work without limitation on publication year was eligible. Data extraction and analysis were guided by a template comprising approaches, methodologies, and tools to translate family systems care into the care continuum. Data are presented in tabular form with an accompanying narrative summary. Our search identified 454 articles, of which three papers matched the inclusion criteria after screening of titles, abstracts, and finally, full texts. The gray literature search yielded 13 findings, of which three were included. Six papers remained for the overall synthesis. Limited evidence exists on approaches and methodologies for implementing family systems care programs within the care continuum in maternal, newborn, and child health in LMICs. A clear, culturally adapted definition of family, essential for advancing research and practice, is lacking. Leadership, facilitation, and attitudinal change influence family systems care program implementation. Existing implementation frameworks need adaptation to the LMICs settings.

## Paper Context

- **Main findings:** Research on family systems care within the maternal, newborn, and child health continuum in low- and middle-income countries remains limited.
- **Added knowledge:** Implementation success is shaped by supportive leadership, facilitation strategies, and healthcare professionals’ attitudinal shifts towards the work with family.
- **Global health impact for policy and action:** Future implementation research can build on existing frameworks, adapting them to the contextual factors that influence family-systems care programs in low- and middle-income countries.

## Background

### Rationale

Availability, affordability, and quality of maternal, newborn, and child healthcare (MNCH) services are critical to reducing mortality, morbidity, and disability [1]. The maternal health package refers to women’s health during pregnancy, childbirth, and the postpartum period [2]. Newborn health includes the care for every newborn, comprising small and sick newborns, from birth up to the first month of life [3]. The child health package contains health services for all children, including those with disabilities and sick or injured children [4].

In MNCH programs, the ‘continuum of care’ (CoC) may have two meanings. The first is the care that is provided as a continuum throughout the lifecycle, including pregnancy, childbirth, childhood, and adolescence. The second meaning is the provision of care on a continuum that includes home, the community, primary care and hospital level [5]. Providing MNCH in a CoC along this second definition could significantly improve MNCH outcomes in low- and middle-income countries (LMICs) [6,7].

An effectively coordinated care process involves and empowers families and places them at the center of the care process [8–10]. Although quality family-centered care is expected to minimize the risk of mortality, morbidity, and disability, families are rarely involved in MNCH services provided at the hospital, community, and home level in LMICs [11–13]. Generally, only mothers and their children are targeted and not the family as a whole [6].

A way to involve families in the caregiving of their newborns and children from pregnancy through birth, postnatal care and childhood, is through family systems care (FSC). FSC facilitates tailored family history assessments, explores needs, and provides ongoing psychosocial support by considering the human being as a physical and mental being within their eco-social living environment [14–16]. FSC is an extension of the concept of family-centered care [17]. Both concepts view families and health providers as mutually beneficial partners revolving around principles of dignity and respect, information sharing, shared decision making, and collaboration [8]. We use the term FSC to refer to all concepts above, including family systems nursing, family-focused care, family-centered care, and family engagement.

Recent studies have shown that women and families with low birth weight infants, premature infants, or sick children need diverse support, including psychosocial, practical (household chores), material, and spiritual to facilitate effective care of their infants in the hospital and at home [18–20]. Health providers, particularly nurses and midwives, are uniquely positioned to provide evidence-based information and psychological support and to empower families to cope with illness and suffering [21–23].

Benefits of FSC have been reported to include reduced stress, anxiety, improved symptoms, and everyday life management for families [24–26]. FSC has proved to increase breastfeeding rates and neonatal weight gain [26–28]. Through FSC, sensitivity in caring for the newborn improved [29,30]. Family systems intervention yielded robust results concerning parental well-being, thereby improving family-child interaction and advancing child development [31].

FSC throughout the CoC aims to provide comprehensive care, addressing medical needs, emotional and social support to families [1]. It ensures that families receive consistent assistance and information from pregnancy through childhood [32]. While this combination has not been extensively explored, it appears crucial for providing holistic care to families throughout the entire CoC.

Translating FSC into practice is complex, and sustaining it is challenging [33,34]. It demands robust research methods and implementation approaches [33,35]. Implementation science uses rigorous methods to identify ‘what works,’ ‘why,’ and ‘how’ to improve health outcomes through evidence-based practices [36]. Implementation science theories, models, and frameworks guide research translation, explore implementation factors, and evaluate effectiveness [37].

Knowledge of implementation strategies of FSC interventions in real-world settings of LMICs remains exceptionally rare [38–40], even more so along the CoC. Therefore, we considered a scoping review to be the most suitable approach to exploring the current state of publications on this topic.

### Objectives of the scoping review

The objective of this scoping review was to identify and map the extent of literature comprehensively and explore approaches, methodologies, and tools used to translate FSC into the CoC of MNCH in LMICs.

Specifically, the review explored across the CoC of MNCH in LMICs:

- **1**: What family systems care (FSC) interventions/programs have been integrated into the continuum of care (CoC) for maternal, newborn and child health (MNCH) in low- and middle-income countries (LMICs) and what approaches, methodologies, and tools have been used?
- **2**: What contextual determinants, including mitigating and limiting factors, affect the uptake of FSC practices and effective translation of interventions/programs across the CoC for MNCH in LMICs?
- **3**: Which family members and health care professionals are involved in FSC interventions/programs across the CoC for MNCH in LMICs?

### Participants

This scoping review included two groups of participants.

The first target group included families of patients using MNCH services. We used the family concept by Shajani and Snell [16] *‘Family is who they say they are’*. Therefore, the family could also be a friend or a neighbor.

Accordingly, we included mothers, fathers, siblings, grandparents, and other relatives or significant caregivers such as friends and neighbors involved in the care process. Families with the target child of the intervention up to 12 years of age, or a woman who used maternal health services, were considered.

The second target group included healthcare providers and lay workers delivering MNCH services. Healthcare providers comprised nurses, midwives, advanced practice nurses and midwives, physician assistants, physicians, as well as lay personnel such as community volunteers and traditional birth attendants delivering some MNCH services.

### Concept

This scoping review explored the concept of family systems care (FSC). It included studies using the following terms: family systems care, family-centered care, family nursing care, family-focused care, family participatory care, and family engagement. All publications that reported on methodologies, approaches, and tools used to translate FSC into MNCH, and contextual determinants that influence this translation were included. Studies of FSC focusing on other areas outside the field of MNCH, such as FSC for adults or elderly people, were excluded.

### Context

To be included in the scoping review, programs or interventions had to occur in the care pathways between hospital, community, and home, including discharge, referral, or transfer procedures along this CoC. Articles were expected to report on the transition between the different care settings. We considered studies conducted in LMICs according to the World Bank [41] definition (full list in Annex I).

## Material and methods

A scoping review approach was chosen due to the broad nature of the research question. It was an ideal tool to determine the scope or coverage of a body of literature, to give an indication of the volume of literature and studies available, and an overview of its focus [42]. Scoping reviews are helpful when the literature in an area is complex, heterogeneous, and not suited to a more focused systematic review [43]. The proposed scoping review was conducted in line with the Joanna Briggs Institute (JBI) methodology for scoping reviews [43]. We used the Preferred Reporting Items for Systematic Reviews and Meta-Analysis extension for Scoping Reviews (PRISMA-ScR) guidelines [44,45].

### Protocol and registration

A scoping review protocol was written according to the PRISMA-ScR [46] and registered prospectively in Open Science Framework on 29 May 2023 [47].

### Eligibility criteria

Following JBI guidance [43], the inclusion and exclusion criteria were categorized and defined in terms of participants, concept, context (PCC), and sources (Table 1) [48]. Published English literature without a time limit until the week two January 2024 was eligible. Gray literature was included to increase the comprehensiveness of available evidence [49], as ongoing programs from non-governmental organizations made public on web pages may not result in scientific publications.

**Table 1.** Eligibility criteria.

Inclusion Criteria	Defining characteristics
Participants	*Family members* using MNCH services, families with children up to the age of 12, women using maternal health services, parents, siblings, grandmothers, grandfathers, uncles, aunts, and other significant caregivers *Healthcare providers*: nurses, midwives, advanced practice nurses and midwives, physician assistants, doctors *Lay personnel*: e.g. community health volunteers, traditional birth attendants delivering Maternal and Child Health services
Concept	Family systems/care/nursing/centered care, family engagement intervention and programs, approaches, methodologies, tools, contextual determinants, including enabling and limiting factors for successful translation
Context	Low- and middle-income countries. Studies should be in care continuum settings, including community (primary care), home and hospital/health facilities
Type of Sources	Any existing literature, including journal articles, gray literature, and information on webpages and evaluation reports.
Time Period	No time restriction
Language	English
Exclusion Criteria	Defining characteristics
Reason for exclusion	Documents written in languages other than English Studies not within the scope of MNCH Studies with a person-centered approach (not family-centered) aiming at one person alone Not meeting the inclusion criteria listed above

MNCH = Maternal, Newborn, and Child Health.

### Information sources

The search strategy was designed to identify published and unpublished primary studies, texts and opinion papers. We followed the recommended three-step approach of Peters, Godfrey [50].

Step 1 consisted of a preliminary search of the Medical Literature Analysis and Retrieval System Online (MEDLINE), the Cumulative Index to Nursing and Allied Health Literature (CINAHL), and the Web of Science Core Collection. This allowed the identification of initial search terms and keywords. We checked all three databases for Medical Subject Headings (MeSH) and Keywords Plus, respectively. We used the RefHunter protocol to define a systematic search string [51] and to document the search. The search strategy, including all identified keywords and indexed terms, was adapted for each included database and information source (Annex II). We used a decision tree to guide the inclusion or exclusion of articles (Annex III).

In step 2, we performed a search using the identified strategy in Medline (Ovid), CINAHL (Ebsco), and Web of Science Core Collection. For the gray literature, available information on 30 organization homepages was searched (Annex IIII). In step 3, the references of retrieved publications and documents were searched for additional suitable literature.

### Search

Keywords and search terms (family-centered care; continuum of care; maternal; newborn; child health; low-and middle-income countries; country names) were used in various forms. MeSH terms and keywords plus Boolean operators (AND/OR) and the asterisk operator (*) to identify variations of the original word were used to create the search string for the different databases. (Annex xx) After completing the first search in the three databases in June (week 3, 2023), a secondary search was performed in December (week 1, 2023) to identify the most recent publications. Organizational homepages were searched between June (week 1, 2023) and January (week 1, 2024). References from all articles included were last searched in January (week 2, 2024).

### Selection of sources of evidence

All identified citations were collated and uploaded into EndNote (Clarivate Analytics, PA, USA version 20.6) and transferred to Rayyan software [52]. Duplicates were removed using its automatic function [52]. The remaining citations were screened by titles and abstracts.

First, two independent reviewers (CS, GEN) conducted a pilot test of 10 articles to assess reviewer agreement regarding the relevance of titles and abstracts. After the pilot phase, potentially relevant sources were retrieved in full, and their citation details were imported into Rayyan software. Two independent reviewers (GEN, STNO) assessed the full text for eligibility criteria. Any disagreements between the reviewers at each stage of the selection process were resolved through discussion with a third reviewer (CS).

The organization’s web references were searched for potentially relevant information according to eligibility criteria using the same keywords and search terms. If titles, subtitles, or texts appeared relevant, references were documented in an Excel file and checked again for inclusion criteria by two reviewers (CS, RB). In case of disagreements in the screening process, an agreement was reached with a third reviewer (GEN).

Within each of these search processes, ‘snowball’ reference searching was applied, a method reported to increase effectively the thoroughness of search results in reviews [53]. Reviewers held regular meetings to discuss and resolve emerging issues, such as unclear content or instances where multiple articles reported on the same study.

## Data charting process

### Data extraction

Data were extracted from papers independently by three reviewers (CS, GEN, RB) using a data extraction tool developed by the reviewers (Table 2). The data extracted included details on study participants, concept, context, study methods, tools used, and key findings relevant to the review questions. The draft data extraction tool was modified and revised as necessary while extracting data. These adjustments reported findings in more detail than the originally drafted table. Any disagreement between reviewers was resolved through discussion until a consensus was reached.

**Table 2.** Characteristics of studies in the scoping review (*n* = 6 studies).

Publication details					Participants		Domain	Context
Authors and year of publication	Way of identification	Title	Journal and DOI	Country of study/program Year of data collection	Type of family members*	Type of health providers		
Maria et al., 2021	Database Search	Assessment of feasibility and acceptability of family-centered care implemented at a neonatal intensive care unit in India	BMC Pediatrics https://doi.org/10.1186/s12887-021-02644-w	India June to July 2016	Mothers, fathers, other family members	Nurses, physicians	Newborn Health	Hospital: from enrolment through discharge, home care
Maria and Agrawal, 2021	Database Search	Family-Centered Care for Newborns: From Pilot Implementation to National Scale-up in India	Indian Pediatrics https://doi.org/10.1007/s13312-021-2358-4	India 2014	Mothers, fathers, grandparents	Nurses, physicians	Newborn Health	Hospital: from enrolment through discharge, home care
Albayrak and Büyügönec, 2022	Database Search	The impact of family-centered care interventions on neonatal and parental outcomes in a Turkish Hospital	Elsevier Collegian https://doi.org/10.1016/j.colegn.2022.05.004	Turkey April 2017 – September 2018	Mothers, fathers	Nurses, physicians, managers, executive nurse, nurse supervisor, nurse educator, researcher, physician supervisor, consultant for families	Newborn Health	Hospital: from enrolment through discharge, with calls after discharge
Tiruneh et al., 2020	Gray (Reference search)	Effectiveness of participatory community solutions strategy on improving household and provider health care behaviors and practices: A mixed-method evaluation	PLoS One https://doi.org/10.1371/journal.pone.0228137	Ethiopia March 2016 – November 2017	Mothers, fathers, grandparents, aunt/uncle	Nurses, midwives, health officers, health extension workers, Women’s development army members	Maternal and Newborn Health	Community, health facilities, health posts
Mhango et al., 2020	Gray (Reference search)	Implementing the Family-Led Care model for preterm and low birth weight newborns in Malawi: Experience of healthcare workers	African Journal of Primary Health Care & Family Medicine (AOSIS) https://doi.org/10.4102/phcfm.v12i1.2266	Malawi March 2017-November 2018	Mothers, fathers, other family members	Nurse-midwives, medical assistants, clinical officers, hospital attendants, community health workers, community volunteers (e.g care groups)	Newborn Health	Hospital: from enrolment through discharge, community and home care
Nanyunia et al., 2022	Gray (Reference search)	Early care and support for young children with developmental disabilities and their caregivers in Uganda: The Baby Ubuntu feasibility trial	Frontiers Pediatrics https://doi.org/10.3389/fped.2022.981976	Uganda Most likely 2020–2021	Mothers, fathers, grandmother, aunt, expert parents	Healthcare worker (not specified)	Child Health	Hospital, community, home care

Publication details				Concept			
Authors and year of publication	Objectives	Research Design	Methods Details	Type of intervention/program	Implementation methodologies/approaches/tools	Enabling factors	Limiting factors
Maria et al., 2021 [54]	1) To examine the feasibility and acceptability of implementing a model in the NICU. 2) To use the evaluation to inform further development of an implementation framework for the FSC model 3) To inform scale-up of this model within India and beyond.	Prospective cohort design	Quantitative data: 1) Demographics parent attendant 2) Training session attendance 3) Parent-attendant activities 4) Unit staff sensitization sessions attendance 4) Staff participation in conducting training sessions for parents	A comprehensive audio-visual training tool with four sequential modules Providers led skill-building daily training sessions for parent-attendants that focused on: Session 1: Sensitization to FSC Session 2: Developmentally Supportive Care Session 3: Kangaroo Mother Care Session 4: Preparation for Discharge and Care at Home Healthcare providers offered: 1) Psychosocial and tangible ongoing support 2) Continuous communication with parent-attendants in the NICU	**Training** *Health providers* 1) Sensitizing sessions (at the beginning of the study/every three months until the end of the study) to the family-centred care approach 2) Training as how to train and support family members 3) In-training session and discussion for healthcare providers **Implementation outcome measures** 4) *Fidelity*: assessed through supervision of sensitization session + training session 5) *Feasibility*: assessed practicality of program implementation, participation rates of healthcare providers in sensitization sessions 6) *Acceptability* by *health providers*: Percentage of all participating healthcare providers who participated in conducting parent-attendant training sessions 7) *Acceptability by families*: measured through percentage of parents who participated in the program trainings and the parent-attendants’ ability to accurately complete program activities No other specific implementation framework mentioned	1) High program fidelity 2) Flexible sensitization sessions 3) Family members’ willingness to participate 4) Program acceptability of healthcare providers and families 5) Favorable family visiting policy at NICU. 6) Improvement of hospital philosophy and strategic priorities relation to FSC 7) Acknowledgement from leadership 8) Engaged healthcare providers and buy-in 9) Staff familiarity and confidence in FSC program 10) Senior nursing officer oversaw all sensitization and training activities 11) Support from NICU medical director	1) Conventional mindset of healthcare providers (position of commanding authority) 2) Low acceptability of interventions (handwashing) 3) Challenge for parents to achieve new skills in short time 4) Rigid visiting rules due to pandemic 5) Turnover of health staff
Maria and Agrawal, 2021 [55]	1) To translate and adapt FSC principles into a feasible, acceptable, and sustainable model of FSC 2) To eventual scale up in the public health system	Implementation study	Not described	Culturally sensitive comprehensive audio-visual training package which includes: 1) Audio-video training 2) Training guide package covering practices related to infection prevention, including hand washing 3) Entry protocol 4) Providing developmentally supportive care 5) Activities of routine care including: − Feeding − Technique of breastfeeding − Expression of breast milk − Assisted feeding of a low-birth-weight baby − Diaper change − Kangaroo mother care 6) Preparing parents and family for discharge and post-discharge care of the high-risk baby at home 7) Continuing essential newborn care practices 8) Recognizing danger signs 9) Timely care seeking in the event of sickness	No specific implementation framework mentioned	1) Basic amenities for mothers/attendants close to the newborn care unit 2) Space for training and conducting counseling 3) Attitudinal change amongst the healthcare providers to accept parents/family as partners and embrace core components of family participation and equal partnership in care 4) Framework outlining care activities for caregivers 5) Partnering with organizations to adapt the FSC model for secondary-level newborn care facilities. 6) Scale up through external funds 7) National operational guideline for scale-up 8) Federal funding support 9) Context-specific adaptation 10) Baseline need-gap assessment 11) Tailoring implementation state-wise 12) Strong leadership by local champions 13) Political commitment 14) Commitment from health facility team, administrators and district and state program managers 15) Investments in health systems 16) Flexible implementation 17) Concerted efforts for health system strengthening 18) Cascaded capacity building 19) Ongoing support for newborn care with a quality assurance component built into the program	1). Risk of over-reliance and task shifting by the conventional care providers to the primary caregivers 2) Lack of sufficient space 3) Inadequate infrastructure for accommodating the caregivers 4) Staff shortages 5) Perceived risk of infection through caregivers 6) Security considerations 6) Interference with workflow 7) Concerns about task-shifting and inability to participate 8) Lack of interest from family members 9) Lack of ‘buy-in’ from HCP 10) Let go of position of authority
Selvinaz Albayrak, Lale Aysegül, Büyügönec, 2022 [56]	1) To improve nurses’ attitudes towards parental engagement 2) To examine the impact of implementing nursing interventions related to family-centered care on neonatal and parental outcomes in a university hospital in Turkey	Quasi-experimental, non-equivalent, Post-test research design	Quantitative data: 1) Preterm Characteristics 2) Parental Characteristics 3) Maternal Attachment Inventory (MAI) 4) Empowerment of Parents in the Intensive Care-Neonatology (EMPATHIC-N) 5) Parent Participation Attitude Scale (PPAS)	Ten FSC nursing interventions: 1) Parents’ orientation to the NICU 2) Providing guidebooks and educational material to parents 3) Implementing an open visitation policy for parents (parent’s visitation hours increased from one hour to 4–6 h/daily with this open visitation policy) 4) Allowing parents at the bedside during interdisciplinary team rounds 5) Educating parents at the bedside about care delivery 6) Observing and assisting mothers for breastfeeding 7) Increasing and ensuring parental involvement in the care of the infant (hygienic care, skin-to-skin kangaroo care, oral feeding, etc.) 8) Coordinating parent support groups meetings (psychologist, lactation consultant, consultant for families of premature infants) 9) Planning the discharge from the time of admission, providing educational materials, and training parents for the discharge process during their hospitalization10) Monitoring the infant’s health after discharge with daily phone calls in the first week, at the end of the second week, and end of the first month.	Implementation Process of FSC Nursing Interventions **Initial Phase** Determining FSC nursing interventions a) Establishing a FSC committee. b) Selecting the ten evidence-based FSC nursing intervention **Phase 1) Preparing for FSC implementation** − Conducting four-hour training session for nurses − Measuring nurses’ attitudes toward FSC with parent participation attitude scale − Meeting with medical staff about implementing FSC nursing intervention − Meeting with support staff about implementing FSC nursing intervention **Phase 2) Training for FSC intervention** − Training nurses and other health team members (four hours) − Attitudes towards FSC re-examined **Phase 3) Implementing FSC nursing intervention** − Performance evaluation in implementing FSC nursing intervention − Continuous monitoring for inadequate performing nurses − Re-evaluation of nurses’ attitudes No other specific implementation framework mentioned	1). Intension of NICU Management to initiate FSC 2). Identifying of problems during implementation and necessary improvement − Organizing the physical environment − Establishing FSC practices − Visiting procedures − Improving staff knowledge, skills, attitudes to increase parental involvement in care − Identifying specific needs regarding the units’ culture − Consideration of opinions from both parents and healthcare team (physicians, nurses, managers) − Continuous training to maintain a positive attitude 7) Facilitator (clinical nurse) for both parents and nurses during FSC nursing interventions 8) Open visiting hours 9) Eliminating visitation restrictions for siblings, parents and relatives 10) Encouraging parental involvement in kangaroo care and hygiene (positive impacts on maternal attachment) 11) Support from NICU physician for NICU nurses 12) Determination of NICU nurses roles in FSC 13) Hospital administrators and nurse managers support in: − research − contribution towards development of procedures − funding provision 14) Encouragement to change attitude towards FSC 15) Supervision of nurses	None mentioned
						16) Counselling services for parents (parent motivation to participate) 17) Provision of recourses 18) Monitorization of outcomes of infants, parent and institution	
Tiruneh, 2020 [57]	1) To implement a participatory community and facility quality improvement intervention, the Participatory Community Solutions (PC-Solutions) 2) To remove supply- and demand-side barriers to desirable maternal and newborn care behaviors and practices	Pre-/post-test non-equivalent group study design	Mixed Method data: 1) Household survey (Maternal and Newborn healthcare indicators 2) in-depth interviews	1) Birth notification: to promote postnatal care 2) Family conversation: a forum conducted at the home of a pregnant woman with her family members and relatives who are encouraged to support the woman during pregnancy, labor, delivery, and the postpartum period to promote birth preparedness and essential newborn care	Four-step quality improvement process (plan-do-study-act) 1) Joint assessment to identify gaps: − workflow mapping − client exit interviews − document review − focus group discussion 2) Meeting with community members, health extension workers, health center staff, woreda health office staff, and referral hospital staff to discuss assessment findings, consolidate points, and identify priority problems and their solutions. 3) Implementation of solutions 4) Joint monitoring − monthly follow-up and coaching − monthly quality improvement meetings at health centers and in communities for health post staff, health extension workers, women, and women’s development army members to review data and progress − Quarterly learning sessions − Quality improvement refresher training for facilitators	1) Full stakeholder participation in all stages of the project 2) Strong coordination 3) Robust support 4) Continuous performance review 5) Staff commitment facilitated the implementation of the PC-Solutions strategy 6) High community engagement in quality improvement planning, implementation, and monitoring 7) Shared responsibilities at all levels of the woreda health system 8) Detailed micro-plan indicating who is responsible for what and when 9) Development of coordinated activities, especially communication between the community, Women development army, health extension workers and health centers 10) Participatory approach involving stakeholders 11) Continuous assessment of the problems and identifying potential solutions, plan accordingly 12) Support system (facilitating review forums, conducting supervision and mentoring from partners and/or woreda health office) 13) Continuous refresher training and supportive supervision visits to monitor and coach	1) Staff turnover at the health centers 2) Workload of the health workers and health extension workers 3) Competing priorities of the health service providers and the women development army 4) Frequent community leader changes 5) Short intervention period
Mhango et al., 2020 [58]	1) To gather evidence on the real-life implementation of the newly developed Family-Led Care model in Malawi. 2) Describe the experience of facility-based health surveillance assistants in delivering Family-Led Care. 3) Exploring: a) Knowledge of preterm and LBW identification for admission to a health facility, referral and discharge, adherence to clinical care standards b) Competence in providing inpatient and follow-up care for preterm and LBW newborns c) Attitudes towards Family-Led Care and associated support, health worker perceptions of mothers and family’s reaction to Family-Led Care. Aim of the project model: 1) To improve the quality of care provided in kangaroo mother care units or corners by strengthening kangaroo mother care practices for preterm and low birth weight babies in health facilities according to the Malawi KMC guidelines 2) To engage parents and other family members in the care of their newborns in the health facility and at home post-discharge 3) To ensure a continuum of care from facility to household.	Mixed-methods design	Mixed Method data: 1) Retrospective record reviews 2) Counseling observations 3) Questionnaires 4) Focus groups	1) Counselling flipbook 2) Take-home leaflet 3) Reminder messages 4) Monitoring forms and checklists on feeding/danger signs for families 5) Kangaroo care monitoring sheet 6) Feeding charts for healthcare providers	**Health Facility (Clinical Care)** 1) Training and capacity building 2) Provision of infrastructure, equipment, and supplies 3) Supervision 4) Quality improvement 5) Strengthening the referral system and follow-up care at home and in the community – The practices pathway (community engagement) **Strengthening the referral system and follow-up care** **Home and community care** 1) Take-home materials for families 2) Orientation in Family-Led Care 3) Community sensitization No other specific implementation framework mentioned	1) Implementation under the leadership of the district health management team 2) Clinical training (on newborn care) and orientation 3) Quality improvement conducted 4) Record keeping strengthened health information system 5) Revisiting country’s Kangaroo (mother) guidelines and other preterm and low birth weight care documents to confirm clarity and completeness of criteria, guidelines and protocols and to consider how to strengthen certain aspects, such as discharge criteria	1) Poor in-facility record-keeping (data collection) 2) Short time frame for implementation 3) No proper monitoring and evaluation system in place (for baseline data) 4) Not possible to implement a before – after research design 5) Tools not sufficiently validated in the pilot 6) Training and orientation revised on the go to accommodate new insights 7) Inadequately pretest training and materials over a short project cycle 8) Literacy of family members levels influencing completion of the monitoring form 9) Cultural views of fathers (caring for babies being mothers’ responsibility) 10) Lack of space for enough privacy for families 11) Increased workload
Nanyunia et al., 2022 [59]	Evaluation of 1) Program feasibility and acceptability for caregivers and healthcare workers 2) Preliminary evidence of impact when compared with standard care 3) Factors important for scale-up 4) Provider costs of implementation.	Randomized, pilot feasibility trial, inclusive of a mixed-methods evaluation	Quantitative and qualitative methods 1) Outcome measure instruments to measure evidence of impact on child and caregiver outcomes 2) In-depth interviews 3) Focus group discussions	The program is divided into eight modules, each lasting 2–3 h, covering: 1) understanding disability 2) positioning and carrying 3) feeding 4) mobilizing 5) communication 6) play 7) everyday activities 8) the child within the community Modules are delivered over 4–6 months, incorporating at least one home visit.	Feasibility, acceptability, impact, and scale-up measures No other specific implementation framework mentioned	1) Early community sensitization by local champions, through radio/television programs 2) Positive and caring attitudes of facilitators toward children with disability and their caregivers 3) Peer support of other caregivers 4) Participatory approach to learning/delivery 5) Community engagement promoted ownership e.g. religious leaders and traditional healers as advocates 6) Active early engagement of male caregivers 7) Positive and caring attitudes of healthcare workers and facilitators toward children with disabilities create a conducive, enabling social environment. 8) Good accessibility of training and materials 9) Incentives including transport reimbursement. 10) Need to embed the program within existing community health systems. 11) Geographical locating of groups at more local community clinics rather than central referral hospitals 12) Coordinator and facilitators being easy to contact, which enhanced attendance and adherence 13) Phone call reminders for sessions	1) Lack of community awareness, superstition around etiology of disability including discrimination, stigma and exclusion 2) Poverty (e.g. travelling to session loss of income) 3) Need to manage expectations around the child’s progress 4) Limited engagement of fathers 5) Overstretched health system 6) Low literacy among caregivers’ challenges use of program material

Other family members/other caregivers = not specified FSC: Family System Care.

NICU: Neonatal Intensive Care Unit.

### Data items

The following descriptive and contextual data items were extracted: publication details (place of identification, author, publication year, etc.), type of evidence source, intervention setting (hospital, community, home), study/program objectives, implementation methodologies including approach/tools/duration, enabling and limiting factors and content of the FSC program or study. Details of study participants, concept focus, and context information were documented.

### Synthesis of results

The descriptive and contextual data were extracted first into Excel and then transferred into Microsoft Word tables. Tables were then imported into Atlas.ti version 24.1 for coding and categorization to facilitate group findings for a systematic report. A narrative summary describing how the results relate to the review’s objectives was generated. As usual for scoping reviews, a systematic appraisal of the methodological quality was not performed [43,48].

## Results

**Figure 1.**
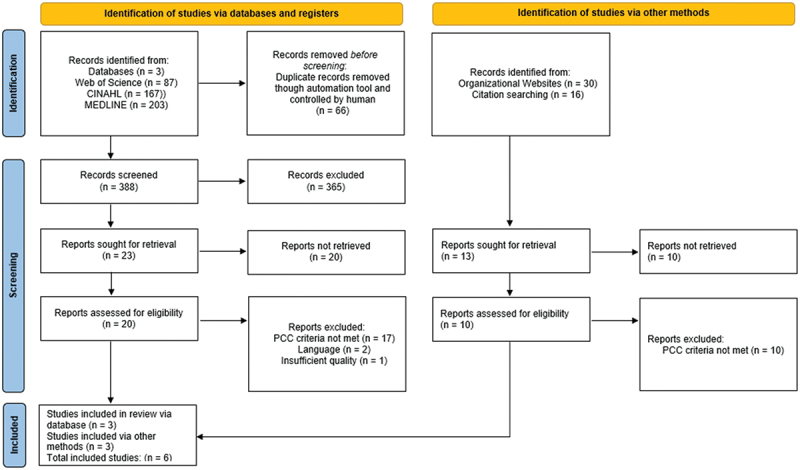
Flow diagram of search and study inclusion process.

Six studies were included in the review (Figure 1), and characteristics are presented in Table 2. The PRISMA-ScR checklist for scoping reviews was used to report results [45] (Annex V).

### Study selection

The search in the databases Medline (Ovid), CINAHL (Ebsco) and Web of Science Core Collection resulted in 454 publications; after removing 66 duplicates, 388 were left. Screening titles and abstracts excluded a further 365, leaving 23 potentially relevant publications. After the full-text assessment, only three matched the inclusion criteria (Figure 1).

The most common reason for exclusion from the studies was the absence of the PCC description, which affected 17 out of 20 papers in this exclusion step. Specific exclusions included: nine studies lacking a focus on implementation, one study not addressing FSC, one study not covering the CoC, and two studies from high-income country settings. Two studies were excluded due to the unavailability of the full text in English and one study was excluded for lacking an implementation focus and involving high-income country settings. Two studies were excluded for multiple reasons: one lacked focus on both implementation and the CoC, and the other included the wrong participants and lacked implementation focus. Although we did not conduct a systematic quality assessment, we excluded one article because its content and methodology was poorly written that it was hardly understandable. The gray literature search on organizational homepages and references of included studies resulted in 13 relevant findings. Three articles that were found through reference searches were included after a detailed assessment. The main reason for exclusion (10 out of 10) was the lack of documentation of the program or implementation process. The review of organizational websites yielded no studies or reports detailing implementation strategies, tools, or methodologies; instead, only a few vague references to implementation steps were available.

## Synthesis of results - summary table of identified papers/approaches

Table 2 summarizes the six identified publications conducted between 2014 and 2018 and published between 2020 and 2022. Results are summarized in the PCC format.

### Participants

#### Type of family members

All six studies included mothers and fathers. Two papers mentioned family members but were not specific about who was included [54,58]. Four reports specifically mentioned one or more family members; three included grandparents [55,57,59], two aunts [57,59], and uncles, and grandmothers [59].

#### Type of healthcare providers

Four papers mentioned nurses [54–57]. Nurse-midwives were reported in one article [58], while another [57] referred to midwives. Three papers included physicians [54–56]. One study [58] included medical assistants, clinical officers, hospital attendants, community health workers, and community volunteers (e.g. care groups). One study [56] mentioned managers, executive nurses, nurse supervisors, nurse educators, researchers, physician supervisors, and consultants for families. Health officers, health extension workers, and women’s development army members were also included [57]. One article [59] mentioned healthcare workers without specifying the type.

### Context

Publications reported single-country studies across three continents: two conducted in Asia (India) [54,55], one in Southeast Europe/West Asia (Turkey) [56], and three in sub-Saharan Africa (Uganda, Malawi, Ethiopia) [57–59]. Two reports from India were from the same project site, but we decided to include them both because they were not conducted during the same period and yielded different findings.

The Ethiopian study [57] focused on the combined maternal and newborn health continuum. Four studies focused purely on newborn health [54–56,58]. The study from Uganda [59] looked at the child health care continuum. All six papers included a hospital- or facility-based care component. Three papers focused on discharge [54–56], and the Turkish study [56] included post-discharge calls without home care, and four included home care [54,55,58,59]. Community care was described in three papers [57–59].

### Concept

#### Objectives

Although all papers focused on families and the care continuum, their objectives differed. Some papers had one primary goal, while others stated several. Although all intended to implement a new intervention program, only three evaluated it. The studies from Turkey and Malawi both examined healthcare providers’ attitudes, though with different emphases [56,58]. The Turkish study [56] focused on promoting family-centered nursing care, while the Malawian study [58] evaluated the implementation and quality of kangaroo care, addressing sub-goals related to healthcare provider competence, knowledge, and attitudes. While one of the studies from India [54] intended to develop an implementation framework for family-centered care model, both studies from India [54,55] evaluated the scale-up process. In Ethiopia [57], the aim was to implement a program, and remove barriers in fostering desirable maternal and newborn care behaviors.

### Research design

Each of the six studies had a different research design. One of the Indian studies [55] used an implementation study design and the other study a prospective cohort design. The authors in Malawi [58] conducted a mixed-method research whiles in Turkey [56] a pre- and post-evaluation was done. Two studies had complex designs: one used a quasi-experimental, non-equivalent design [57] and another employed a randomized pilot feasibility trial [59].

### Methods details

Several overlapping methods were mentioned. The Turkish study [56] described parental and newborn characteristics, whereas one Indian study [54] reported only parental characteristics.

Two studies [58,59] used focus group discussions to collect data. The Ugandan study [59] conducted separate focus groups with female caregivers and healthcare workers to examine feasibility, acceptability, impact, and scale-up. Two studies [57,59] collected data through individual interviews and one [58] used observation techniques. Five studies applied quantitative measures [54,56–59]. One of the studies from India [55] did not report on methodical details.

### Type of methodologies and implementation approaches used

The reviewed studies included various methods and approaches to implementing family-centered care. In the Ethiopian study [57] the research team applied a framework in the form of the ‘Plan Do Study Act’ (PDSA) participatory community quality improvement cycle.

In India [54] training for families was offered. Four studies provided in-service training sessions for healthcare providers [55–58]. The train-the-trainer approach was employed in four studies [54,56–58]. The Ugandan [59] and one of the Indian studies [54] reported implementation outcomes such as feasibility, acceptability, impact, and scaling-up. The latter study [54] assessed fidelity through supervision of sensitization and training sessions. Feasibility was measured through the rates of healthcare providers conducting sensitization sessions acceptability of FSC for health providers was tested during parent-attendant training sessions and for families attending and completing program activities, demonstrating that the approach was acceptable to both groups.

Authors in Ethiopia [57] outlined a combined assessment to identify FSC implementation gaps through workflow mapping, client exit interviews, document review, and focus group discussions. Performance evaluation in FSC was critical for the study in Turkey [56]. In the Malawian study supervision, quality improvement, community sensitization, and provision of materials was considered as crucial aspects of FSC implementation and strengthening of the referral system and follow-up care [60].

### Enabling factors

All six articles reported the following overlapping enabling factors for the implementation of FSC.

Acceptance was mentioned as one of the main enabling factors by one of the Indian studies [54]. This attitudinal change meant that health providers accepted families as partners in the CoC of their small or sick infants. Acceptance was defined as recognizing and supporting the core idea of FSC: building a partnership with parents in the care process [55]. Positive and caring attitudes of healthcare workers toward children with disabilities, as well as creating an enabling social environment for families along the care continuum, were mentioned [59]. Acceptance was facilitated by sensitization to FSC, which entailed introducing parents to the Neonatal Intensive Care Unit (NICU) [54] and advocacy for shared responsibility across all levels of the health system [57].

Commitment to FSC from the health facility team, administrators, district and state program managers [55], and stakeholders [57] was considered a strong enabler. Three papers (45–47) mentioned team support coupled with peer support as facilitators.

Five papers reported supportive leadership as a determining factor for an optimal FSC program [54–58]. The intention of the NICU management to initiate FSC [56] and political commitment [55] enabled smooth implementation of FSC.

The presence of a clinical nurse facilitator was considered an essential enabling factor for both parents and nurses. Easy to contact coordinators and facilitators were seen as beneficial for attendance and adherence to FSC [56,59]. Facilitation included forum reviews, supervisions, and mentoring from partners, thus providing a sense of belonging and a feeling of appreciation [56,57].

High community engagement was essential in the quality improvement planning, implementation, and monitoring of FSC [57,59]. Among some community stakeholders, such as religious leaders and traditional healers, community engagement was seen to promote ownership of FSC [57,59].

Baseline assessment prior to implementation was highlighted as supporting a program implementation [55,56] by identification of hurdles early. Baseline assessments entailed the identification of specific needs of a unit culture and the consideration of opinions from both families and the healthcare team [55]. It involved assessment of training material [55] and a clear program description [56]. Context-specific adaptation was mentioned as enabling [55].

The importance of program monitoring and evaluation was considered an enabling factor in implementing FSC by four authors [55–58]. Aspects of monitoring included outcomes of infants, parents, and institutions as well as continuous screening for nurse performance [56]. Additional proposed monitoring aspects were monthly progress follow-up, coaching, and monthly quality improvement meetings at health centers and communities for health post staff, health extension workers, and women’s development army members [57]. Review checklists were mentioned as helpful [55,58].

Well-laid-out guidelines to foster the scaling up of the intervention were considered enabling. A national operational guideline, for instance, revealed the importance of scale-up [55]. Revisiting national kangaroo care guidelines and other guidelines on preterm and low birth weight care to improve clarity, citing the example of discharge criteria was suggested [58]. Three studies indicated that outlining care activities was an essential enabling factor for the implementation of FSC [55–57]. Coordinating activities and communication between parties involved, such as the community, women’s development army, health extension workers, and health centers, was considered supportive of the success of FSC [57].

Skill training and capacity building for improved motivation were considered critical enabling factors put forward more specifically by two papers [55,56]. The provision of basic resources [56] and the flexibility of the implementation process [55] were also considered important.

### Limiting factors

The selected papers also highlighted several limiting factors to FSC implementation, including high staff turnover and shortage, over-reliance and task shifting, poverty, and inadequate infrastructure. Diverse cultural expectations, stigma, and community challenges were additional limitations.

High staff turnover at health centers [54,57], competing priorities of different service providers [57], and frequent interference in the workflow were limiting factors reported. Staff shortages [55] and increased workload of health workers and health extension officers were pointed out [57,58].

Lack of buy-in from healthcare providers and low acceptability of interventions were described as limiting factors [54,55]. The conventional mindset of healthcare providers (position of commanding authority) was pinpointed as a significant barrier [54]. Maria and Agrawal [55] also raised concerns about healthcare providers becoming over-reliant, leading to a task shifting to the primary caregivers.

High expectations [59] or, on the lack of interest from family members limited, in some cases, the overall implementation of FSC [55,59]. The Malawian study [58] identified a lack of community awareness and cultural views, notably by fathers considering caring for babies an exclusive maternal responsibility, as a limiting factor. One study [59] mentioned superstition and stigma concerning disability. Frequent changes in the community leader was identified as a significant limiting factor to the implementation of FSC programs [57].

Inadequate pretest training and materials over a short project cycle, resulting in improvised training [58], and the short time frame of the implementation period [54,57] were important limiting factors to the FSC program’s implementation.

Poverty [59] and low literacy levels of family members negatively influenced the completion of monitoring forms and the use of program material, limiting the implementation of the FSC [58,59]. Inadequate infrastructure to accommodate caregivers, lack of space for family privacy [55,58], and rigid visiting rules, particularly during the pandemic, limited implementation of FSC programs [54].

### Type of interventions and programs

The six included studies presented a range of interventions with various contents and focuses, although they remained related to family-centered care. The two Indian papers of Maria and Agrawal [55] and Maria, Litch [54] originated from the same program using audiovisual tools.

Maria, Litch [54] described four sequential modules: sensitization, developmentally supportive care, kangaroo care, as well as discharge preparation with post-discharge home care. Maria and Agrawal [55] provided more detailed program content, highlighting nine key interventions, including routine care activities, essential newborn care, and preparing families for discharge and home-based care for high-risk infants, emphasizing timely care-seeking.

The Turkish study [56] detailed ten nursing interventions, such as providing educational material, implementing open visitation policies, breastfeeding support, and promoting parental involvement in infant care. It also included discharge planning in collaboration with parents, and phone support during the first month at home.

In Ethiopia, the authors [57] aimed to promote antenatal and postnatal care through pregnancy and birth notification done by the health extension workers and family conversations, a forum conducted at the home of the pregnant women with family members to encourage support throughout pregnancy, labor, delivery, and postpartum, with a focus on birth preparedness and essential newborn care.

The study from Malawi [58] used monitoring forms and checklists as essential elements of their FSC program, such as kangaroo care monitoring sheets, feeding charts, and danger sign checklists for families and healthcare providers.

The Ugandan study [59] built the ubuntu-hub program [61] with eight modules for families of children with disabilities, covering topics such as understanding disability, daily activities, communication with a disabled child, interaction with healthcare providers, and the child’s rights within the community.

## Discussion

### Summary of evidence

Our findings highlight the scarcity of research on family-centered care within the care continuum for maternal, newborn, and child health in LMICs. Only six papers included FSC implementation along the care continuum with outlined implementation details.

### Families

Although the retrieved studies involved various family members, foremost parents, none provided a clear definition of ‘family’. Previous research highlighted the lack of clear definition of ‘family’ in LMICs [39,62,63]. For programs in LMICs to be effective, they must consider the collectivist cultures and extended families’ influence on pregnant women and children, particularly regarding decision-making [64,65] A Eurocentric definition of family may hinder program success, so understanding the local cultural context and definition of ‘family’ is crucial before program design. We argue that establishing a clear, culturally adapted definition of family is critical to ensure research integrity, support replication, and facilitate the successful translation of interventions into practice [66]. A precise definition of ‘family’ facilitates research replication and addresses obstacles in advancing clinical practice, care enhancement, and outcome improvement [66,67].

None of the studies described a specific method to identify or strategically assess family members or support persons, as proposed by Wright and Leahey [24]. Understanding patient perceptions of their family structure and its functions is essential for strategically involving families in family systems care (FSC). This understanding informs research, practical applications, interventions, and policy development [67]. Tools like genograms and ecomaps can effectively assess family structures, supporting the networks and facilitating an organized transition between levels of care [68]. This systemic approach considers individuals, family relationships, and their connections with health providers, the healthcare system, society, and culture. This approach is well-suited to maternal, newborn, and child health, as it systemically addresses challenges and resources within families and their contexts [69].

### Health providers

The six studies highlighted a large variety of healthcare providers, most frequently nurses, midwives, physicians, and particularly community health volunteers. Other reviews from LMICs support the effectiveness of community health volunteers in ensuring a strong CoC, especially in rural areas [70,71]. Including them in the family-centered CoC for maternal, newborn, and child health care appears essential.

### Methodologies

No study utilized determinants, implementation or process frameworks. For future programs, we strongly recommend training and capacity-building programs to support structured implementation frameworks.

Implementation tools adapted for LMICs are limited. To date, only two have been modified for use in LMICs. One is the Community Context Assessment for Community Health (COACH) tool by Bergström, Skeen [72]; the other one is the Consolidated Framework for Implementation Research (CFIR) [73]. Means, Kemp [74] evaluated the CFIR and recommended additional domains and constructs to increase its compatibility for LMICs that contrast with high-income countries by differing facilitators and hurdles. In a nutshell, implementation research tools need further adaptation to the collectivistic settings that are common in LMICs settings [75,76].

Only one study [57] included an implementation approach: the Plan-Do-Study-Act (PDSA) cycle. The World Health Organization specifically recommends the PDSA cycle as a quality improvement strategy for maternal and newborn health projects both in health facilities and at the community level [77–79].

Implementation outcome measures are recommended to report key elements such as acceptability, adoption, appropriateness, feasibility, fidelity, implementation cost, penetration, and sustainability alongside contextual information [80–82] and effectively guide the translation of programs from theory into practice [83,84]. Despite these recommendations, only two of the six studies identified reported any implementation outcome measures [54,59].

### Interventions

Our review identified family-centered care programs mainly focusing on neonatal health, neglecting maternal health, older infants with disabilities, the antenatal period, family planning, older children with chronic conditions/palliative care, and perinatal loss along the CoC.

The family-centered care interventions in the few available studies were diverse in their objectives, research designs, and implementation approaches. This diversity of interventions contributes to the complexity of implementing family-centered care programs [80].

Three studies addressed developmental care interventions in newborns and older children. Although research on developmental care is receiving increasing attention, it remains very scarce in LMICs [85–87]. Family-centered and developmental care for children has proven to enhance cognitive, motor, and language outcomes; it focuses on more than survival and providing an environment where children can thrive [88].

### Enabling and limiting factors

All six included studies reported on a wide range of contextual factors except for one [56], which did not report on limiting factors. Phiri, Chan [38] found that family-centered care implementation for hospitalized children is poorly documented due to inconsistencies, limiting insights into facilitators and barriers – a challenge echoed in our search, where we had to exclude nine articles lacking implementation details.

### Enabling factors

Most papers mentioned improved staff knowledge, skill development, and attitudinal change as helpful in implementing and increasing family involvement in health care. Although few publications on newborn and child health report healthcare providers’ attitudes toward families [38,89,90], studies detailing characteristics associated with healthcare providers’ positive attitudes toward families are lacking [91]. These studies would be crucial as healthcare providers’ attitudes toward patients and families in maternal, newborn, and child health are often considered worrying [92–95]. Healthcare providers need training in family-centered care along the care continuum, including outside research projects. Only funding for consistent and systematic training can support local ownership [96].

Although supervision and facilitation were mentioned as facilitating factors in some of the included papers, no detailed description was given. Facilitation is considered one of the vital components in translating intervention into practice [97–99]. Bergström, Hoa [77] suggest that these factors are likely to enhance the participation and value for all involved. Further work is needed to understand what types of supervision and facilitation are effective and how they can support the implementation of family-focused care along the care continuum in MNCH in LMICs.

In our scoping review, the allowance of only a short implementation time frame was considered a hindrance to successful implementation. Time factors are often related to financial constraints but must be incorporated when planning and implementing new interventions.

### Limiting factors

Insufficient participant buy-in or engagement with the innovation (here, FSC) can pose a substantial barrier to successful implementation [100]. This lack of buy-in may stem from limited acceptability or a poor fit between the innovation’s design and the participants’ needs, priorities, or contextual circumstances [101]. Conducting a thorough context analysis and assessing interest in the innovation are key steps before rolling out any new, evidence-based interventions. Training and sensitization sessions for healthcare providers should be integral to an implementation endeavor to increase interest and buy-in [100]. If context analysis reveals considerable barriers or limited interest, implementers should adapt either the contextual factors or the innovation itself through a participatory approach involving stakeholders such as healthcare professionals, managers, and families.Comparison of our findings with those of other studies confirms that a shortage of health providers and high staff turnover rates, including increased workload, are generally considered to negatively impact family-centered care uptake [102–104]. High staff turnover in LMICs represents a significant challenge, as it not only creates a continuous demand for training new staff but also places an additional burden on the remaining healthcare workers. It has been suggested that good family care can be provided even under time constraints, as a 15-minute family interview may involve families in purposeful, effective, and efficient care [68,105].

Appropriate funding was identified as key for successful implementation and scale-up. As much as in-country or government funding is necessary, research relies heavily on outside donors and continues to be inconsistently funded (81). Despite its impact on newborn survival, implementation and neonatal care research receives limited funding [106], while support for nursing and midwifery science – vital for advancing family care – remains unclear.

Several limiting and enabling factors regarding policy and organizational support were mentioned. Hospitals with more open and flexible visiting hours, for instance, significantly enabled the uptake of family-centered care [107]. A potential barrier to family involvement in NICUs is the concern about infection risks, further highlighted during the COVID pandemic [108]. However, none of the studies included in our review reported this directly, although one described infection-prevention sessions.

In our review, the availability of guidelines and policies was considered contributing to success, although family-centered care guidance is often missing in actual procedures, guidelines, and policies [80].

### Implications

A significant factor contributing to the limited number of publications on the implementation methods of FSC in LMICs is the consistently low funding allocated for both implementation and newborn research [106]. This gap is of concern for advancing evidence-based practices in family-centered care, particularly in maternal, newborn, and child health and LMICs. However, it would likely increase maternal, newborn and child survival [106].

In-depth assessments of the local context and health system function are essential (88). The successful integration of FSC requires meticulous planning of medium – and long-term projects and the development of comprehensive documentation to support wider adoption [80]. We concur with previous research proposing collaboration between policymakers, researchers, and healthcare providers to prioritize implementation science locally and globally [76,80].

### Strength and limitations

Our scoping review has several strengths. The review team employed a comprehensive consensus process to establish study inclusion/exclusion criteria, and a standardized screening and data extraction process using the Rayyan software. Scrupulously following the PRISMA-ScR [45] and JBI [43] guidance strengthened the quality of our review. Our review included an extensive search of organizational homepages alongside the three databases (Medline, CINAHL, Web of Science).

Several limitations must be considered. Some publications may not have been included in our review if only using the term ‘caregiver’ as we preferred using more specific, non-ambiguous terms from antenatal to postnatal care. While we aimed for comprehensive coverage, we may have missed documents on organizational homepages or in conference proceedings. Since homepage searches were resource-intensive and, after thirty organizations, did not yield any relevant results, we limited the search accordingly. Using PubMed instead of Medline may have broadened inclusion for emerging or gray literature. Overall, as scoping reviews by design do not assess the methodological quality of included studies, our results remain suggestive rather than conclusive.

## Conclusions

Publications on family-systems care implementation approaches and methodologies along the care continuum of maternal, newborn, and child health in LMICs remain very limited. Current evidence cannot recommend a specific methodology or implementation tool for LMICs. Rather than developing new approaches, we suggest that future research focuses on existing implementation frameworks, tools, and approaches used in high-income countries and adjusts these to a low- and middle-income context. This scoping review highlights similarities and trends among enabling and hindering factors. Important factors were adopting attitudinal change through training, facilitation, and capacity building, as well as allocating sufficient time for implementation. Supportive leadership and available policies and guidelines contribute to implementation success. A clear, culturally adapted definition of ‘family’ and a detailed description of the implementation process are crucial for improving research and practice when implementing family-systems care programs.

## Supplementary Material

Annex IIII_Organizational Homepages_20250215.docx

Annex I_List_LMICs_20250215.docx

Annex V_PRISMA_ScR_Checklist_20250215.pdf

DecisionTree_Revision1_20250822.pdf

Annex II_SearchStrategy_20250215.docx

## Data Availability

All articles included in this review are listed in Table 2 and are listed in the reference list. The complete set of data generated during this study are available from the corresponding author on reasonable request. Please also see appendix I-IV in the Supplementary Data.

## Review registration

Registered at Open Science Framework (https://osf.io/zwg9k).
