# Supplementary material for: Family systems care approaches and methodologies for maternal, newborn and child health in low- and middle-income countries: a scoping review
Source: Glob Health Action. 2025 Oct 15;18(1):2567714. doi: 10.1080/16549716.2025.2567714 (PMC12529736; doi:10.1080/16549716.2025.2567714)
Supplement: Annex IIII_Organizational Homepages_20250215.docx [file ZGHA_A_2567714_SM1117.docx]

### File IIII: Organizational homepages

| **Organizations** | | |
| --- | --- | --- |
| African Medical and Research Foundation (AMREF) | Care International | Enfants du Monde |
| Family Health International 360 (FHI 360) | Healthy Newborn Network (HNN) | Inter-Agency Working Group on Reproductive Health in Crises (IAWG) |
| IBP Network | International Committee of the Red Cross (ICRC) | International Rescue Committee (IRC) |
| Johns Hopkins Program for International Education in Gynecology and Obstetrics (Jhpiego) | John Snow, Inc. (JSI) | Management of At-risk Mothers and Infants under six months (MAMI) Global Network |
| Marie Stopes International (MSI) | Médecins Sans Frontières (MSF) | Newborn Essential Solutions and Technologies 360 (NEST 360) |
| newborntoolkit.org | PATH | Population Council |
| RAISE Initiative | Results for Development (R4D) | Resilient, Inclusive, Sustainable Education (RISE 360) |
| Save the Children | Solidarmed | SickKids for Global Health |
| United Nations International Children's Emergency Fund (UNICEF) | United Nations Population Fund (UNFPA) | USAID Momentum |
| Women’s Refugee Commission (WRC) |  |  |
