# Supplementary material for: Family systems care approaches and methodologies for maternal, newborn and child health in low- and middle-income countries: a scoping review
Source: Glob Health Action. 2025 Oct 15;18(1):2567714. doi: 10.1080/16549716.2025.2567714 (PMC12529736; doi:10.1080/16549716.2025.2567714)
Supplement: Annex I_List_LMICs_20250215.docx [file ZGHA_A_2567714_SM1116.docx]

### File I: List of LMICs

AFGHANISTAN

ALBANIA

ALGERIA

AMERICAN SAMOA

ANGOLA

ARGENTINA

ARMENIA

AZERBAIJAN

BANGLADESH

BELARUS

BELIZE

BENIN

BHUTAN

BOLIVIA

BOSNIA AND HERZEGOVINA

BOTSWANA

BRAZIL

BULGARIA

BURKINA FASO

BURUNDI

CABO VERDE

CAMBODIA

CAMEROON

CENTRAL AFRICAN REPUBLIC

CHAD

CHINA

COLOMBIA

COMOROS

CONGO, DEM. REP.

CONGO, REP.

COSTA RICA

COTE D'IVOIRE

CUBA

DJIBOUTI

DOMINICA

DOMINICAN REPUBLIC

ECUADOR

EGYPT, ARAB REP.

EL SALVADOR

EQUATORIAL GUINEA

ERITREA

ESWATINI

ETHIOPIA

FIJI

GABON

GAMBIA, THE

GEORGIA

GHANA

GRENADA

GUATEMALA

GUINEA

GUINEA-BISSAU

GUYANA

HAITI

HONDURAS

INDIA

INDONESIA

IRAN, ISLAMIC REP.

IRAQ

JAMAICA

JORDAN

KAZAKHSTAN

KENYA

KIRIBATI

KOREA, DEM. PEOPLE'S REP.

KOSOVO

KYRGYZ REPUBLIC

LAO PDR

LEBANON

LESOTHO

LIBERIA

LIBYA

MADAGASCAR

MALAWI

MALAYSIA

MALDIVES

MALI

MARSHALL ISLANDS

MAURITANIA

MAURITIUS

MEXICO

MICRONESIA, FED. STS.

MOLDOVA

MONGOLIA

MONTENEGRO

MOROCCO

MOZAMBIQUE

MYANMAR

NAMIBIA

NEPAL

NICARAGUA

NIGER

NIGERIA

NORTH MACEDONIA

PAKISTAN

PALAU

PAPUA NEW GUINEA

PARAGUAY

PERU

PHILIPPINES

RUSSIAN FEDERATION

RWANDA

SAMOA

SAO TOME AND PRINCIPE

SENEGAL

SERBIA

SIERRA LEONE

SOLOMON ISLANDS

SOMALIA

SOUTH AFRICA

SOUTH SUDAN

SRI LANKA

ST. LUCIA

ST. VINCENT AND THE GRENADINES

SUDAN

SURINAME

SYRIAN ARAB REPUBLIC

TAJIKISTAN

TANZANIA

THAILAND

TIMOR-LESTE

TOGO

TONGA

TUNISIA

TURKIYE

TURKMENISTAN

TUVALU

UGANDA

UKRAINE

UZBEKISTAN

VANUATU

VIETNAM

WEST BANK AND GAZA

YEMEN, REP.

ZAMBIA

ZIMBABWE

[Low & middle income | Data (worldbank.org)](https://data.worldbank.org/income-level/low-and-middle-income) 31.05.2023
