# Supplementary figures and images for: Family systems care approaches and methodologies for maternal, newborn and child health in low- and middle-income countries: a scoping review
Source: Glob Health Action. 2025 Oct 15;18(1):2567714. doi: 10.1080/16549716.2025.2567714 (PMC12529736; doi:10.1080/16549716.2025.2567714)

File II: Decision Tree

1

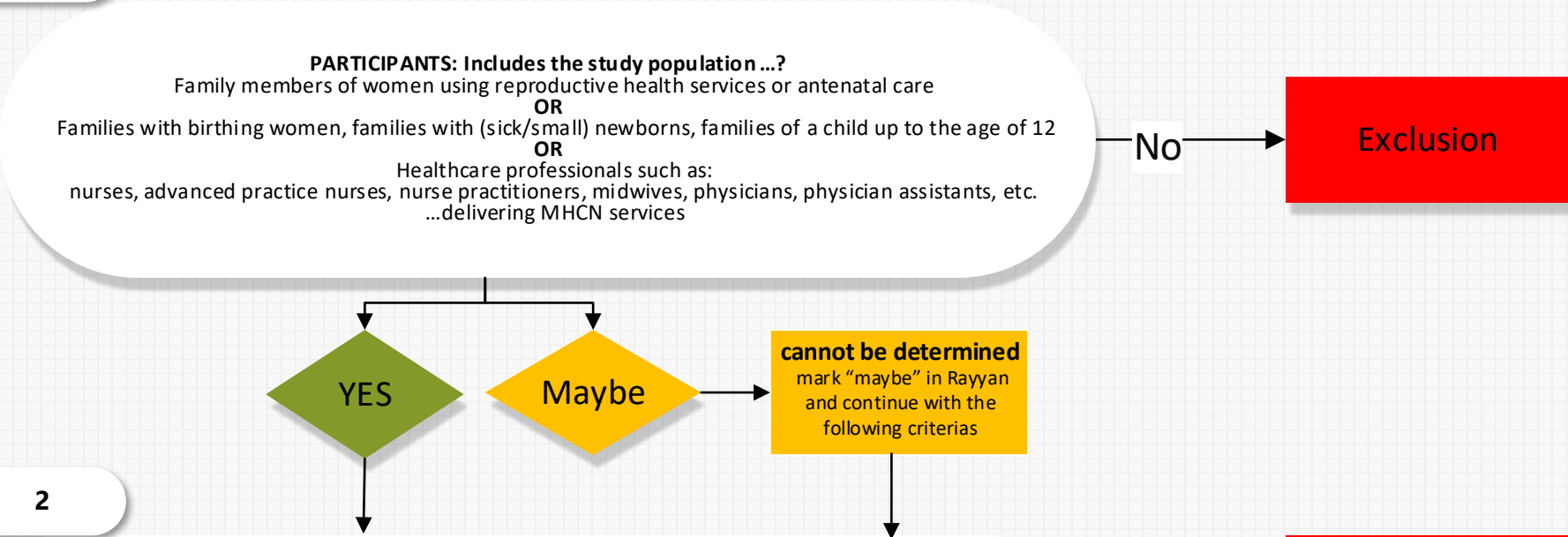

2

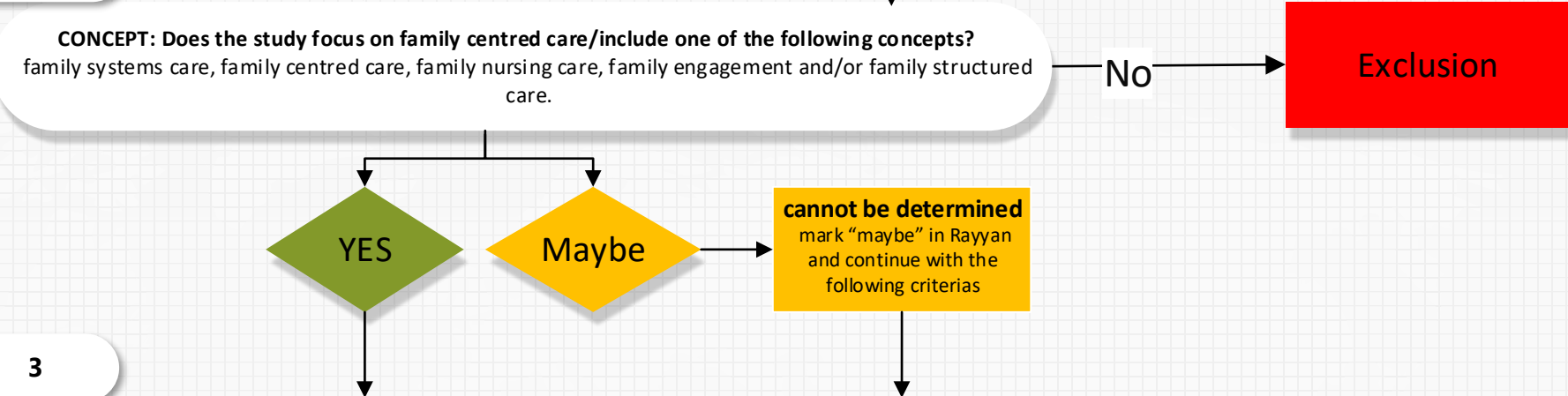

3

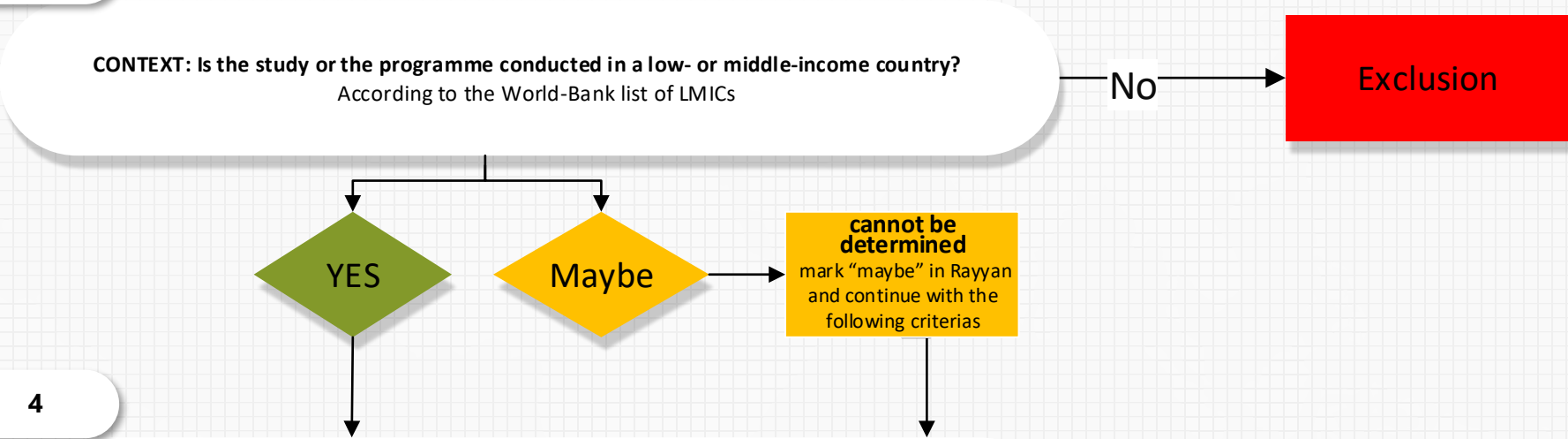

4

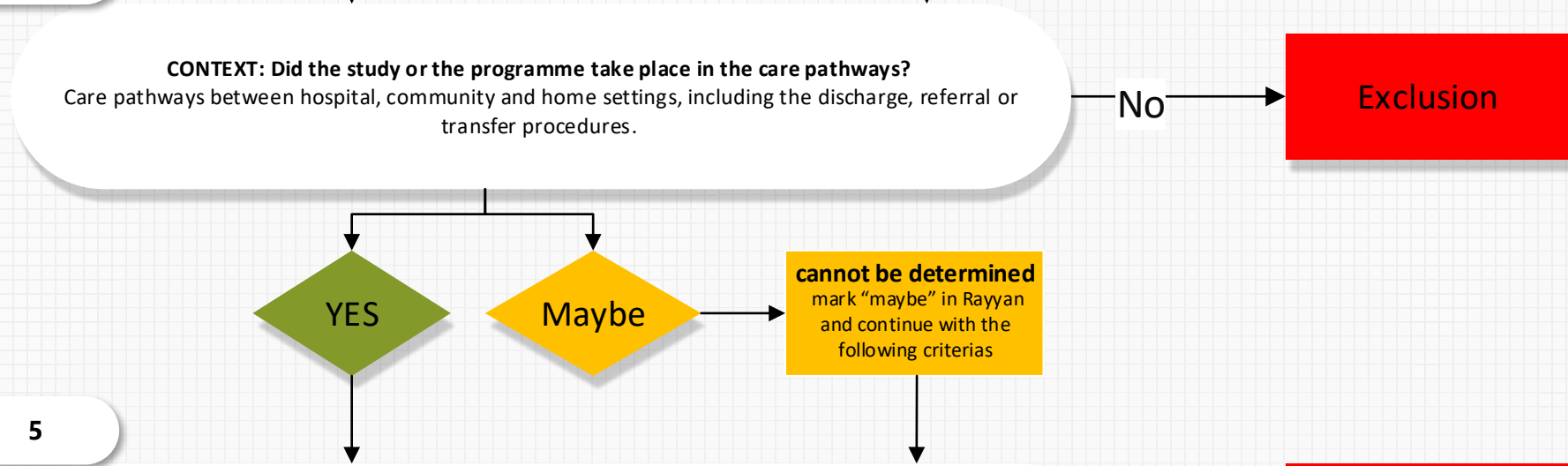

5

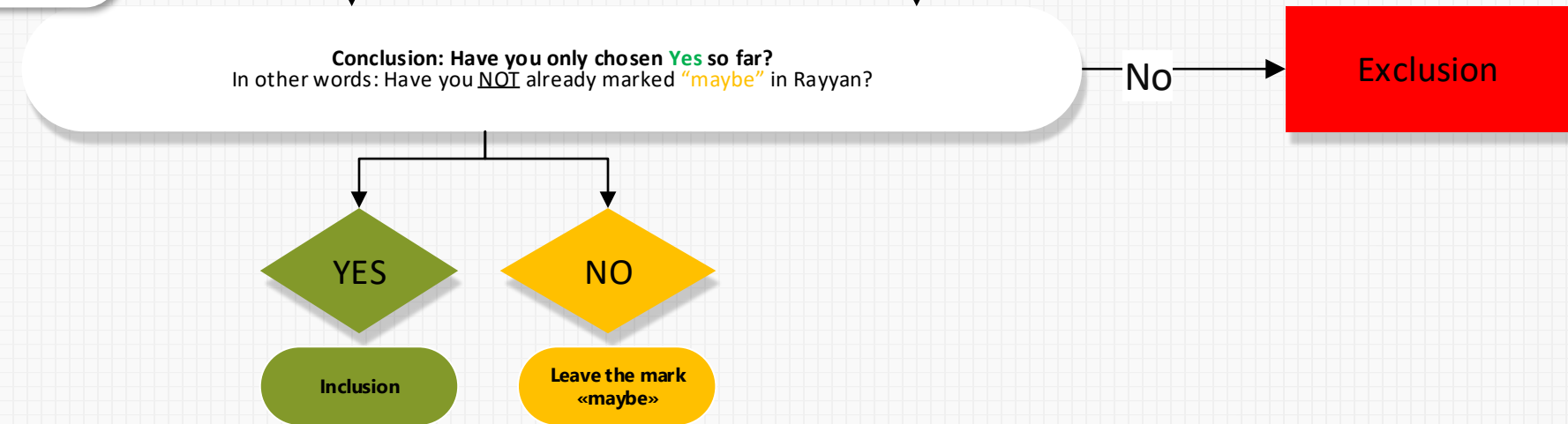

6

For detailed contextual assessment

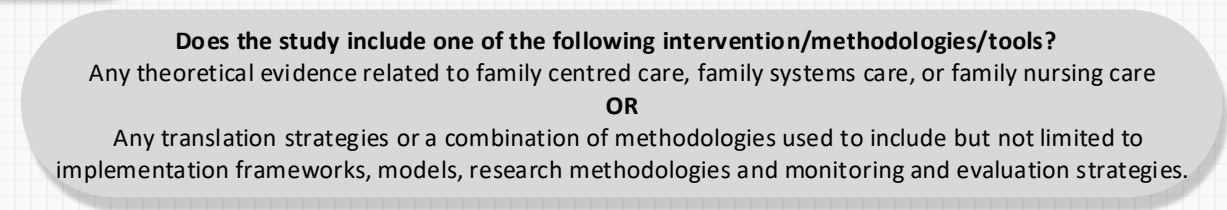

Supplement: DecisionTree_Revision1_20250822.pdf [file ZGHA_A_2567714_SM1114.pdf]
