# Supplementary material for: Family systems care approaches and methodologies for maternal, newborn and child health in low- and middle-income countries: a scoping review
Source: Glob Health Action. 2025 Oct 15;18(1):2567714. doi: 10.1080/16549716.2025.2567714 (PMC12529736; doi:10.1080/16549716.2025.2567714)
Supplement: Annex II_SearchStrategy_20250215.docx [file ZGHA_A_2567714_SM1113.docx]

### File II: Search strategy

((

"FAMILY adj2 *CENTRED* adj2 *CARE*" OR

"FAMILY adj2 *CENTERED* adj2 *CARE*" OR

"FAMILY" OR

"FAMILIES" OR

"FAMILY adj2 *NURSING*" OR

"FAMILY adj2 *CARE* adj2 *NURSING*" OR

"FAMILY adj2 *FOCUSED* adj2 *CARE*" OR

"FAMILY adj2 *ORIENTED* adj2 *CARE*" OR

"FAMILY adj2 *SYSTEMS* adj2 *NURSING*" OR

"FAMILY adj2 *SYSTEMS* adj2 *APPROACH*" OR

"FAMILY adj2 *CENTERED* adj2 *CARE* adj2 *INTERVENTION*" OR

"FAMILY adj2 *CENTRED* adj2 *CARE* adj2 *INTERVENTION*" OR

"FAMILY adj2 *PARTICIPATORY* adj2 *CARE*" OR

"FAMILY adj2 *SUPPORT*"

).mp

OR

(

exp FAMILY NURSING OR

exp FAMILY SUPPORT OR

exp FAMILY

).sh)

AND

((

"CARE* adj2 *CONTINUUM*" OR

"CONTINUITY adj2 OF adj2 CARE*" OR

"CARE* adj2 CONTINUITY" OR

"PATIENT* adj2 CARE* adj2 CONTINUITY" OR

"CARE* adj2 PATHWAY*" OR

"*REFERRAL* adj2 PATHWAY*" OR

"*REFERRAL* adj2 *COMPLETION*" OR

"*DISCHARGE*" OR

"PATIENT* adj2 DISCHARGE*" OR

"PATIENT* adj2 HANDOFF*" OR

"CONTINUOUS adj2 *CARE*" OR

"TRANSITIONAL adj2 *CARE*"

).mp

OR

(

exp CONTINUITY OF PATIENT CARE OR

exp HOSPITAL TO HOME TRANSITION OR

exp PATIENT DISCHARGE OR

exp PATIENT HANDOFF OR

exp TRANSITIONAL CARE

).sh)

AND

((

"MATERNAL adj2 HEALTH*" OR

"PRENATAL* adj2 *CARE*" OR

"ANTENATAL* adj2 *CARE*" OR

"INTRAPARTUM* adj2 *CARE*" OR

"PREGNANCY" OR

"MATERNAL* adj2 *CARE*" OR

"POSTPARTUM* adj2 *PERIOD*" OR

"CHILD* adj2 BIRTH*" OR

"BIRTH*"

).mp

OR

(

exp MATERNAL HEALTH OR

exp MATERNAL HEALTH SERVICES OR

exp PREGNANCY OR

exp PERINATAL CARE

).sh)

AND

((

"HEALTH* adj2 OF adj2 NEWBORN* adj2 INFANT*" OR

"HEALTH* adj2 OF adj2 THE adj2 NEWBORN* adj2 INFANT*" OR

"NEWBORN* adj2 INFANT* adj2 HEALTH*" OR

"BABY adj2 HEALTH*" OR

"BABIES adj2 HEALTH*" OR

"NEONATAL* adj2 HEALTH*" OR

"NEWBORN* adj2 HEALTH*" OR

"POSTNATAL* adj2 *CARE*" OR

"NEWBORN* adj2 *CARE*" OR

"PRETERM* adj2 INFANT*" OR

"NEWBORN* adj2 INFANT*" OR

"NEONATAL* adj2 INTENSIVE* adj2 *CARE*" OR

"PREMATURITY" OR

"LOW adj2 BIRTH* WEIGHT*"

).mp

OR

(

exp INFANT OR

exp INFANT HEALTH OR

exp INFANT CARE OR

exp PRENATAL CARE OR

exp INFANT, NEWBORN OR

exp INFANT, NEWBORN, DISEASE OR

exp INFANT, LOW BIRTH WEIGHT OR

exp INFANT, PREMATURE OR

exp INFANT WELFARE

).sh)

AND

((

"CHILDREN* adj2 *HEALTH*" OR

"CHILD* adj2 *CARE*" OR

"CHILDREN" OR

"CHILD*" OR

"CHILD* adj2 WELL BEING*" OR

"CHILD* adj2 WELLBEING*" OR

"CHILD* adj2 HEALTH* adj2 SERVICE*" OR

"YOUNG* adj2 CHILDREN*"

).mp

OR

(

exp CHILD OR

exp CHILD HEALTH OR

exp CHILD CARE OR

exp CHILD WELFARE OR

exp CHILD-HEALTH SERVICES OR

exp MATERNAL-CHILD HEALTH SERVICES OR

exp MATERNAL-CHILD HEALTH CENTERS

).sh)

AND

((

"DEVELOPING adj2 COUNTRIES" OR

"DEVELOPING adj2 NATIONS" OR

"DEVELOPING adj2 NATION*" OR

"LEAST* adj2 DEVELOPED adj2 COUNTRY" OR

"LESS adj2 DEVELOPED adj2 COUNTRY" OR

"LESS adj2 DEVELOPED adj2 COUNTRIES" OR

"THIRD* adj2 WORLD* adj2 COUNTRIES" OR

"THIRD* adj2 WORLD* adj2 COUNTRY" OR

"THIRD* adj2 WORLD* adj2 NATION*" OR

"UNDER-DEVELOPED adj2 COUNTRY" OR

"UNDERDEVELOPED adj2 COUNTRIES" OR

"UNDERDEVELOPED adj2 COUNTRY" OR

"UNDERDEVELOPED adj2 NATION*" OR

"LOWER adj2 MIDDLE adj2 INCOME adj2 COUNTRY" OR

"LOWER-MIDDLE-INCOME adj2 COUNTRIES" OR

"LOW adj2 INCOME adj2 COUNTRIES" OR

"LOW adj2 INCOME adj2 COUNTRY" OR

"MIDDLE adj2 INCOME adj2 COUNTRY" OR

"MIDDLE adj2 INCOME adj2 COUNTRIES" OR

"DEVELOPING adj2 WORLD*" OR

"LESSER adj2 DEVELOPED-COUNTRIES" OR

"RURAL adj2 AREA*" OR

"SUBSAHARAN AFRICA*" OR

"SOUTHEAST ASIA" OR

"SOUTH AMERICA*"

).mp

OR

(

exp DEVELOPING COUNTRIES OR

exp RURAL HEALTH SERVICES OR

exp RURAL HEALTH OR

exp RURAL HOSPITALS OR

exp ASIA, SOUTHEASTERN OR

exp AFRICA SOUTH OF THE SAHARA OR

exp SOUTH AMERICA

).sh

OR

(AFGHANISTAN OR ALBANIA OR ALGERIA OR "AMERICAN SAMOA ANGOLA" OR ARGENTINA OR ARMENIA OR

AZERBAIJAN OR BANGLADESH OR BELARUS OR BELIZE OR BENIN OR BHUTAN OR BOLIVIA OR "BOSNIA AND

HERZEGOVINA" OR BOTSWANA OR BRAZIL OR BULGARIA OR "BURKINA FASO" OR BURUNDI OR "CABO VERDE" OR

CAMBODIA OR CAMEROON OR "CENTRAL AFRICAN REPUBLIC" OR CHAD OR CHINA OR COLOMBIA OR COMOROS

OR CONGO OR COSTA RICA OR "COTE D'IVOIRE" OR CUBA OR DJIBOUTI OR DOMINICA OR "DOMINICAN REPUBLIC"

OR ECUADOR OR EGYPT OR "ARAB REPUBLIC" OR "EL SALVADOR" OR "EQUATORIAL GUINEA" OR "ERITREA" OR

ESWATINI OR ETHIOPIA OR FIJI OR GABON OR GAMBIA OR GEORGIA OR GHANA OR GRENADA OR GUATEMALA OR

GUINEA OR "GUINEA-BISSAU" OR GUYANA OR HAITI OR HONDURAS OR INDIA OR INDONESIA OR IRAN OR "ISLAMIC

REPUBLIC" OR IRAQ OR JAMAICA OR JORDAN OR KAZAKHSTAN OR KENYA OR KIRIBATI OR KOREA OR "DEM*

PEOPLE'S REP*" OR KOSOVO OR "KYRGYZ REP*" OR "LAO PDR" OR LEBANON OR LESOTHO OR LIBERIA OR LIBYA

OR MADAGASCAR OR MALAWI OR MALAYSIA OR MALDIVES OR MALI OR "MARSHALL ISLANDS" OR MAURITANIA OR

MAURITIUS OR MEXICO OR MICRONESIA OR "FED* STS*" OR MOLDOVA OR MONGOLIA OR MONTENEGRO OR

MOROCCO OR MOZAMBIQUE OR MYANMAR OR NAMIBIA OR NEPAL OR NICARAGUA OR NIGER OR NIGERIA OR

"NORTH MACEDONIA" OR PAKISTAN OR PALAU OR "PAPUA NEW GUINEA" OR PARAGUAY OR PERU OR PHILIPPINES

OR "RUSSIAN FEDERATION" OR RWANDA OR SAMOA OR "SAO TOME AND PRINCIPE" OR SENEGAL OR SERBIA OR

"SIERRA LEONE" OR

"SOLOMON ISLANDS" OR SOMALIA OR "SOUTH AFRICA" OR "SOUTH SUDAN" OR "SRI LANKA" OR "ST. LUCIA" OR

"SANTA LUCIA" OR "ST. VINCENT AND THE GRENADINES" OR SUDAN OR SURINAME OR "SYRIAN ARAB REP*" OR

SYRIA OR TAJIKISTAN OR TANZANIA OR THAILAND OR "TIMOR LESTE" OR TOGO OR TONGA OR TUNISIA OR TURKIYE

OR TURKMENISTAN OR TUVALU OR UGANDA OR UKRAINE OR UZBEKISTAN OR VANUATU OR VIETNAM OR

"WEST BANK AND GAZA" OR YEMEN OR ZAMBIA OR ZIMBABWE

).mp

)
